# CHOP regulated by METTL14-m6A affects cell cycle arrest and regorafenib sensitivity in HCC cells

**DOI:** 10.1186/s12885-024-12275-w

**Published:** 2024-04-25

**Authors:** Yipeng Pan, Bo You, Xue Zhao, Shanxin Zhang, Wei Li

**Affiliations:** 1 Department of Transplantation, The Second Affiliated Hospital of Hainan Medical University, Haikou, 570100 China; 2 Department of Transplantation, The Hainan General Hospital, Haikou, 570100 China; 3 Department of Ultrasound Medicine, Zhejiang Provincial People’s Hospital, Zhejiang, 311100 China

**Keywords:** HCC, METTL14-m6A, CHOP, Cell cycle, Regorafenib

## Abstract

**Background:**

Regorafenib, a multi-targeted kinase inhibitor, has been used in the treatment of Hepatocellular carcinoma (HCC). The purpose of this study is to investigate the mechanism of Regorafenib in HCC.

**Methods:**

Regorafenib’s impact on the sensitivity of HCC cells was assessed using CCK8. Differential gene expression analysis was performed by conducting mRNA sequencing after treatment with Regorafenib. The m6A methylation status of CHOP and differential expression of m6A methylation-related proteins were assessed by RIP and Western Blot. To explore the molecular mechanisms involved in the therapeutic effects of Regorafenib in HCC and the impact of METTL14 and CHOP on Regorafenib treatment, we employed shRNA/overexpression approaches to transfect METTL14 and CHOP genes, as well as conducted in vivo experiments.

**Results:**

Treatment with Regorafenib led to a notable decrease in viability and proliferation of SK-Hep-1 and HCC-LM3 cells. The expression level of CHOP was upregulated after Regorafenib intervention, and CHOP underwent m6A methylation. Among the m6A methylation-related proteins, METTL14 exhibited the most significant downregulation. Mechanistic studies revealed that Regorafenib regulated the cell cycle arrest in HCC through METTL14-mediated modulation of CHOP, and the METTL14/CHOP axis affected the sensitivity of HCC to Regorafenib. In vivo, CHOP enhanced the anticancer effect of Regorafenib.

**Conclusion:**

The inhibition of HCC development by Regorafenib is attributed to its modulation of m6A expression of CHOP, mediated by METTL14, and the METTL14/CHOP axis enhances the sensitivity of HCC to Regorafenib. These findings provide insights into the treatment of HCC and the issue of drug resistance to Regorafenib.

**Supplementary Information:**

The online version contains supplementary material available at 10.1186/s12885-024-12275-w.

## Introduction

Hepatocellular carcinoma (HCC), referred to as liver cancer, ranks as the second most prevalent cancer globally and is marked by a high recurrence rate and high mortality rate [1–4]. Chronic infection of hepatitis B, fatty liver, obesity, smoking, and alcohol consumption are known risk factors for liver cancer [5, 6]. Treatment approaches for HCC include surgical resection, liver transplantation, chemotherapy, and immunotherapy [7, 8]. However, HCC is often diagnosed at advanced stages, where surgical and chemotherapeutic interventions have limited efficacy, and the prognosis is poor, with potential for metastasis [7]. HCC treatment also includes drug therapies such as Sorafenib, which has been shown to increase the likelihood of survival for patients with advanced HCC, and Lenvatinib, which has demonstrated the potential to enhance the chances of survival for patients with unresectable HCC [9, 10]. Nevertheless, Sorafenib can lead to side effects, including diarrhea and hypertension, while Lenvatinib treatment may be associated with thyroid toxicity concerns [11, 12]. Therefore, it is necessary to identify new therapeutic targets.

Regorafenib is a type II kinase inhibitor [13]. By inhibiting the activity of vascular endothelial growth factor receptor (VEGFR) and RAF proto-oncogene serine/threonine-protein kinase (RAF), Regorafenib effectively blocks cell proliferation and angiogenesis [14, 15]. Regorafenib has demonstrated significant anti-tumor activity in a series of preclinical models, and studies have shown its effectiveness in patients who experience HCC reappearance following liver transplantation [15–17]. However, there is limited research on the mechanism of action of Regorafenib in HCC, and our understanding of its mechanism remains incomplete. Therefore, further investigation into the mechanism of action of Regorafenib in HCC is necessary.

C/EBP-homologous protein (CHOP), referred to as DNA injury-inducible transcript 3 (DDIT3), is a 29kD protein and a significant marker of endoplasmic reticulum stress, which can induce cell cycle arrest [18–20]. Evidence supports the role of CHOP in regulating the hypoxic mechanism in liver cancer cells [21]. Moreover, we discovered an upregulation of CHOP expression through mRNA sequencing analysis following the administration of Regorafenib. Additionally, Regorafenib intervention has been shown to trigger apoptosis and cell cycle arrest in cancer cells [17]. Therefore, we speculate that Regorafenib and CHOP may be involved in the cell cycle arrest process in HCC.

N6-methyladenosine (m6A) is one of the most abundant modifications in mammalian messenger RNAs (mRNAs) and is considered a promising target for diagnosis and therapeutic intervention in cancer [22–25]. m6A modification is regulated by proteins termed as “writer,” “eraser,” and “reader.” [26]. Of these, the primary mediator responsible for m6A modification is the m6A methyltransferase enzyme (writer), such as METTL14 [27, 28]. According to reports, METTL14 has been shown to promote the decay of CHOP mRNA through m6A methylation modification [22]. Furthermore, METTL14 has been found to exhibit an inhibitory effect in HCC and colorectal cancer [27, 29]. We observed a downregulation in the expression of METTL14 after the administration of Regorafenib. However, it is still unclear whether Regorafenib affects HCC through the modulation of the m6A mechanism mediated by METTL14.

To validate this speculation, we first screened Regorafenib-sensitive HCC cells. Subsequently, these selected cells were injected subcutaneously into mice after Regorafenib intervention, while also transfecting them with oe-CHOP, to investigate the impact of Regorafenib and CHOP on tumor growth. Furthermore, we investigated the detailed mechanisms of the actions of Regorafenib, CHOP, and METTL14-m6A in the selected HCC cells, providing valuable insights for the treatment of HCC.

## Materials and methods

### Cell culture

The four HCC cell lines, Huh-7 (AW-CCH089), SK-Hep-1 (AW-CCH036), HCC-LM3 (AW-CCH036), and HepG2 (AW-CCH024), were all purchased from Abiowell. Huh-7 cells and HCC-LM3 cells were cultivated in Dulbecco’s modified Eagle medium (DMEM, D5796-500ML, Sigma, USA), and SK-Hep-1 and HepG2cells were cultivated in a minimal essential medium (MEM, 11,095,080, Gibco, USA). These media were supplemented with 10% fetal bovine serum (FBS, 10,099,141, Gibco, USA) and 1% Penicillin/Streptomycin (AWI0070a, Abiowell, China). Cells were cultured in a saturated humidity incubator (DH-160I, SANTN, China) with 5% CO_2_ at 37℃. The sh-METTL14, oe-METTL14, sh-CHOP, and oe-CHOP plasmids, along with their respective control plasmids, were procured from HonorGene. They were transfected into SK-Hep-1 and HCC-LM3 cells by using Lipofectamine 2000 (11,668,019, Invitrogen, USA).

### Cell grouping and treatment

Group 1 consists of SK-Hep-1 and HCC-LM3 cells that were treated with different concentrations of Regorafenib (0, 5, 10, 15, 30, 60 µM) for 24 h to assess the anti-tumor activity of Regorafenib (S86421, Shyuanye, China) [14]. Group 2 consists of three subgroups: Control, vehicle, and Regorafenib. The two cells were cultured under normal conditions. In the vehicle group, 0.5 µL of DMSO was added to the culture medium of the two cells. In the Regorafenib group, the two cells were exposed to a concentration of 10 µM Regorafenib for 24 h. Group 3 consists of four subgroups: sh-NC, sh-METTL14, oe-NC, and oe-METTL14. In each subgroup of the two cells, transfection was performed with sh-NC, sh-METTL14, oe-NC, and oe-METTL14, respectively. Group 4 consists of four subgroups: vehicle + oe-NC, vehicle + oe-METTL14, Regorafenib + oe-NC, and Regorafenib + oe-METTL14. Group 5 consists of four subgroups: vehicle + sh-NC, vehicle + sh-CHOP, Regorafenib + sh-NC, and Regorafenib + sh-CHOP. Group 5 consists of four subgroups: Regorafenib + Control, Regorafenib + sh-NC, Regorafenib + sh-CHOP, Regorafenib + oe-NC, Regorafenib + oe-CHOP. Group 5 consists of four subgroups: Regorafenib + oe-NC + oe-NC, Regorafenib + oe-NC + oe-CHOP, Regorafenib + oe-METTL14 + oe-NC, Regorafenib + oe-METTL14 + oe-CHOP.

### Cell counting kit-8 (CCK8) assay

Cells were digested using trypsin (AWC0232, Abiowell, China) and cultured at 37℃. After cell attachment, they were treated according to the group requirement for 24 h. Next, 100 µL of medium containing 10% CCK8 reagent (NU679, DOJINDO, Japan) was utilized to replace the drug-containing medium. Finally, the cells were incubated for an additional 4 h before measuring the optical density (OD) at 450 nm using a multifunctional microplate reader (MB-530, HEALES, China).

### 5-Ethynyl-2’-deoxyuridine (EdU) staining

The logarithmically growing cells were seeded onto a well plate and then were treated according to the requirements of each group for 24 h. Following the guidelines provided by the EdU detection kit (C10310, RIBOBIO, China), cells in each group were subjected to EdU labeling, immobilization, Apollo staining, and DNA staining, and images were collected by fluorescence microscope (BA410T, Motic, Singapore).

### Colony formation assay

The two cells (SK-Hep-1 and HCC-LM3) were seeded onto plates, and cultured for two weeks in a humidified incubator at 37℃ with 5% CO_2_. Following PBS wash, each well was treated with 1 mL of 4% paraformaldehyde (N1012, NCM Biotech, China) solution for cell fixation. Following that, 1 mL of crystal violet (G1062, Solarbio, China) was introduced and allowed to incubate at room temperature for 30 min to facilitate staining. Finally, the stained cells were photographed and counted.

### Flow cytometry (FCM)

To conduct cell cycle detection, the two cells were treated with 1.2 mL of pre-cooled 100% ethanol, resulting in a final concentration of 75%. Subsequently, the cells were fixed by overnight incubation at 4℃. After the cells were washed with PBS, they were centrifuged. Then, they were mixed with 150 µL of propidium iodide (PI, MB2920, Meilune, China) and stained for 30 min at 4℃. Different stages of the cell cycle have different DNA content, and PI can label DNA to determine which cycle the cell is in. Detection of stained cells with a flow cytometer (A00-1-1102, Beckman, USA), PI was excited by a 488 nm argon laser and detected through a 630 nm bandpass filter, collecting around 15,000 cells in FSC/SSC dot plots, and analyzing the percentage of cells in each cell cycle phase on the PI fluorescence histogram to determine positive cell staining. For apoptosis detection, SK-Hep-1 and HCC-LM3 cells were treated with trypsin (AWC0232, Abiowell, China). Following digestion, after washing the cells with PBS, approximately 3.2 × 10^5^ cells were collected for further analysis. Following the instruction provided by the apoptosis detection kit (KGA1030, KeyGEN BioTECH, Nanjing, China), the cell suspension was treated with Annexin V-FITC (5 µL) and PI (5 µL) successively. The mixture was then incubated at room temperature for 10 min in the absence of light before further analysis.

### RNA-sequencing (RNA-seq)

Total RNA extraction from SK-Hep-1 cells, HCC-LM3 cells, and tumor tissues was performed using Trizol reagent (15,596,026, Thermo, USA). cDNA was obtained by reverse transcription using a reverse transcription kit (CW2569, CWBIO, China). Subsequently, proceed with the steps of RNA library preparation, sequencing, and data analysis.

### Real-time fluorescence quantitative polymerase chain reaction (RT-qPCR)

Total RNA extraction from SK-Hep-1 cells, HCC-LM3 cells, and tumor tissues was treated with Trizol reagent (15,596,026, Thermo, USA). Reverse transcription was performed by using an mRNA reverse transcription kit, followed by RT-qPCR with UltraSYBR Mixture (CW2601, CWBIO, China). The gene expression was quantified using the 2^−ΔΔCt^ method using β-actin as the internal reference. Detailed primer sequences can be found in Table 1.

**Table 1:** Primer sequences

Gene	Sequences	Length (bp)
CHOP	F GCCCTCACTCTCCAGATTCCA	134 bp
	R TTTCTCCTTCATGCGCTGCT	
METTL14	F GTAGCACAGACGGGGACTTC	195 bp
	R TTGGTCCAACTGTGAGCCAG	
β-actin	F ACCCTGAAGTACCCCATCGAG	224 bp
	R AGCACAGCCTGGATAGCAAC	

### RNA immunoprecipitation (RIP)

The cell pellet (approximately 100 µL) was mixed gently by pipetting with an equal volume of pre-configured RIP Lysis buffer (RIP-12RXN, Sigma, USA) and incubated on ice for 5 min before storing at -80 °C. Subsequently, 50 µL of magnetic beads (20,164, Thermo, USA) were added to each centrifuge tube labeled with IP and Normal IgG. After adding 500 µL of RIP Wash Buffer to the tubes, they were briefly vortexed and centrifuged using a vortex mixer. Then, the centrifuge tubes were placed in the magnetic field to discard the supernatant. The magnetic beads were resuspended in 100 µL of RIP Wash Buffer in each tube, followed by the addition of 5 µg of antibody and thorough mixing. The tubes were rotated at room temperature for 30 min, followed by brief centrifugation and removal of the supernatant using the magnetic field. This washing step was repeated twice. Finally, 500 µL of RIP Wash Buffer was added to each tube, briefly vortexed, and placed on ice. The RNA Immunoprecipitation Kit (RIP-12RXN, Sigma, USA) was utilized following the instructions provided. RNA was purified using TRIzol (15,596,026, Sigma, USA), and cDNA was generated utilizing the mRNA reverse transcription kit with mRNA as a template. The resulting products were used for RT-qPCR experiments.

### Western blot (WB)

Extract total proteins from cells and tumor tissues using Radio immunoprecipitation assay (RIPA) lysate (AWB0136, Abiowell, China). After separation by SDS-PAGE, the protein was tranferred to the nitrocellulose (NC) membranes. Following the blocking step using 5% skimmed milk (AWB0004, Abiowell, China) for 1.5 h, the NC membranes were subjected to incubation with primary antibodies at 4℃ overnight. The NC membranes were subsequently incubated with secondary antibodies. The NC membranes were subjected to incubation with Super ECL Plus detection reagent (AWB0005, Abiowell, China) to facilitate chemiluminescence imaging. The gray values of bands were read by Quantity One 4.6.6 (Bio-Rad Inc., USA). Finally, protein expression was calculated with β-actin as the internal reference. Detailed information for the primary and secondary antibodies can be found in Table 2. Full uncropped Blots images are shown in Figure S4, Figure S5, Figure S6, Figure S7, Figure S8, Figure S9.

**Table 2:** The information on antibody

Name	Dilution rate	Cat number	Source	Company	Country
METTL14	1: 1000	ab300104	Rabbit	Abcam	UK
METTL3	1: 1000	15073-1-AP	Rabbit	Proteintech	USA
ALKBH5	1: 5000	16837-1-AP	Rabbit	Proteintech	USA
FTO	1: 2000	27226-1-AP	Rabbit	Proteintech	USA
CHOP	1: 1000	15204-1-AP	Rabbit	Proteintech	USA
CDK2	1: 1000	10122-1-AP	Rabbit	Proteintech	USA
CDK4	1: 4000	11026-1-AP	Rabbit	Proteintech	USA
CyclinD1	1: 10,000	26939-1-AP	Rabbit	Proteintech	USA
Ki67	1: 50	Ab16667	Rabbit	Abcam	UK
β-actin	1: 5000	66009-1-Ig	Mouse	Proteintech	USA
HRP goat anti-mouse IgG (H + L)	1: 5000	AWS0001	/	Proteintech	USA
HRP goat anti- Rabbit IgG (H + L)	1: 5000	AWS0002	/	Proteintech	USA
CoraLite488-conjugated Affinipure Goat Anti-Rabbit IgG (H + L)	1: 100	SA00013-2	/	Proteintech	USA

### Tumor formation in Nude mice

15 male BALB/c nude mice were purchased at 4 weeks of age from SJA Laboratory Animal Co., Ltd. in Hunan Province, China. After one week of acclimatization, the nude mice were randomly divided into 5 groups (*n* = 3): Control, vehicle + oe-NC, vehicle + oe-CHOP, Regorafenib + oe-NC, and Regorafenib + oe-CHOP. The Control group was injected with untreated SK-Hep-1 cells. The vehicle + oe-NC group and Regorafenib + oe-NC group were injected with SK-Hep-1 cells transfected with oe-NC. vehicle + oe-CHOP group and Regorafenib + oe-CHOP group were injected with SK-Hep-1 cells transfected with oe-CHOP. The vehicle + oe-NC group and Regorafenib + oe-NC group were injected with 2 × 10^6^ SK-Hep-1 cells transfected with oe-NC. The injection volume was 100 µL, and the injection site was the right axillary region. After tumor establishment, tumor volume was measured and observed twice a week. On the 17th day after tumor establishment, intervention was initiated. The Regorafenib + oe-NC group and Regorafenib + oe-CHOP group were intraperitoneally injected with regorafenib (10 mg/kg). The vehicle + oe-NC and vehicle + oe-CHOP groups were intraperitoneally injected with an equal volume of regorafenib solvent (10 mL/kg). The Control group was intraperitoneally injected with an equal volume of physiological saline. The intervention was performed every 2 days for a total duration of 20 days. On the 38th day after tumor establishment, pentobarbital sodium was used to anesthetize mice (at a dose of 50 mg/kg body weight). Then the tumors were collected, and measurements for tumor mass and volume were conducted. Tumor volume is determined by applying the formula: tumor volume = (longest axis) × (shortest axis) × (shortest axis) / 2. Finally, euthanasia was performed by cervical dislocation on the anesthetized mice, to minimize their pain as much as possible. All animal experimental procedures were approved by the ethical committee of the Hunan SJA Laboratory Animal Co., Ltd.

### Immunofluorescence (IF) staining

The harvested tumors were fixed in 4% paraformaldehyde for subsequent paraffin embedding and sectioning, to be used for immunofluorescence staining analysis. The sections were deparaffinized in xylene and dehydrated in a graded ethanol series (75–100%). Subsequently, the sections were immersed in EDTA buffer (pH 9.0) and boiled for antigen retrieval. The sections underwent a sequential treatment process, including immersion in a sodium borohydride solution, followed by 75% ethanol, a Sudan Black dye solution, and finally followed by rinsing with tap water. The sections were blocked with 5% BSA for 1 h and then incubated overnight at 4℃ with the Ki67. Next, 100 µL of was secondary antibodies added and incubated for 1.5 h. Nuclei staining was performed with 4’,6-diamidino-2-phenylindole (DAPI) reagent (AWI0331a, Abiowell, China) for 20 min. The slices were sealed using buffered glycerol (AWI0178a, Abiowell, China) and examined under a fluorescence microscope. Detailed information for the primary and secondary antibodies can be found in Table 2.

### Statistical analysis

The experimental data were subjected to analysis using GraphPad Prism 8.0 software. The data is presented as mean ± standard deviation (SD). To compare multiple groups, we utilized the one-way analysis of variance (ANOVA) followed by Tukey’s posthoc test. We conducted a statistical analysis using a two-way ANOVA with Bonferroni post hoc test to compare groups at different time points. A significance level of *P* < 0.05 was used.

## Results

### Inhibition of HCC proliferation by Regorafenib

**Fig. 1:**
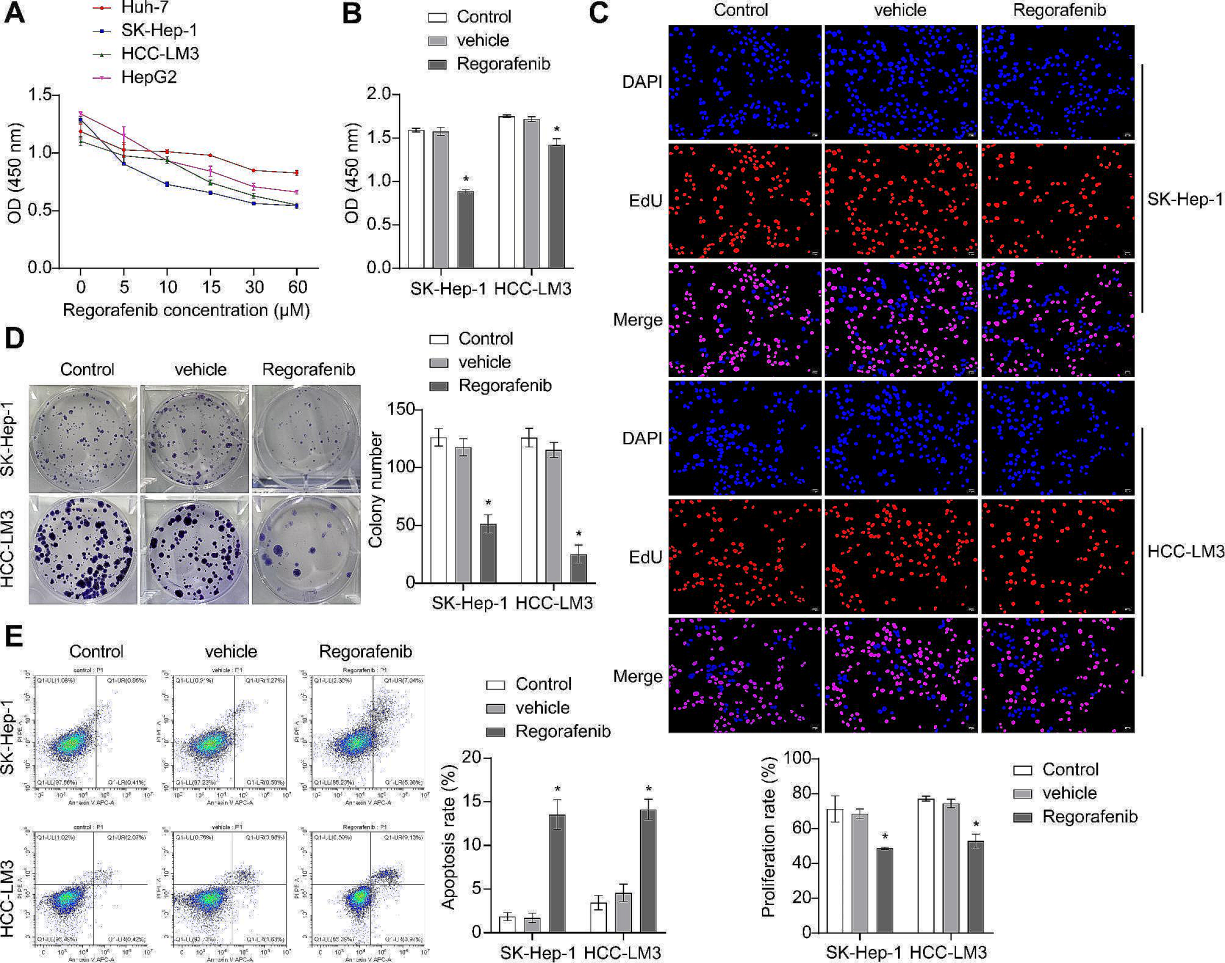
Inhibition of HCC proliferation by Regorafenib. (**A**) CCK8 Assay for the screening of Regorafenib-Sensitive HCC lines and determination of optimal intervention concentration of Regorafenib. (**B**) Determination of the activity of SK-Hep-1 and HCC-LM3 cells using the CCK8 assay. (**C**) Assessment of the proliferation rate of SK-Hep-1 and HCC-LM3 cells using EdU staining. (**D**) Evaluation of the proliferative activity of SK-Hep-1 and HCC-LM3 cells using the colony formation assay. (**E**) Detection of the apoptosis rate in SK-Hep-1 and HCC-LM3 cells using FCM. *P < 0.05 vs. Vehicle

To screen Regorafenib-sensitive HCC cell lines and determine the appropriate intervention concentration of Regorafenib, we treated four types of HCC cell lines (Huh-7, SK-Hep-1, HCC-LM3, and HepG2) with gradient concentrations of Regorafenib (0, 5, 10, 15, 30, and 60 µM) for 24 h. We then assessed the cell viability after treatment using the CCK8 method. As the concentration of Regorafenib increased, the viability of all four HCC cell lines decreased. Among them, the SK-Hep-1 and HCC-LM3 cells exhibited a greater decrease in viability. Through calculations, the IC50 of Regorafenib in HCC cells was determined to be 10 µM. Based on the above results, we selected the SK-Hep-1 and HCC-LM3 cell lines as experimental subjects, with an intervention concentration of 10 µM for Regorafenib (Fig. 1A). After treatment with 10 µM of Regorafenib for 24 h, there was a notable decrease in their proliferative activity. This led to a decrease in the formation of clones and an increase in the number of cells undergoing apoptosis (Fig. 1B and E). The aforementioned results indicate that Regorafenib could inhibit the proliferative activity of HCC cells.

### Regorafenib intervention upregulates CHOP mRNA expression in HCC cells

**Fig. 2:**
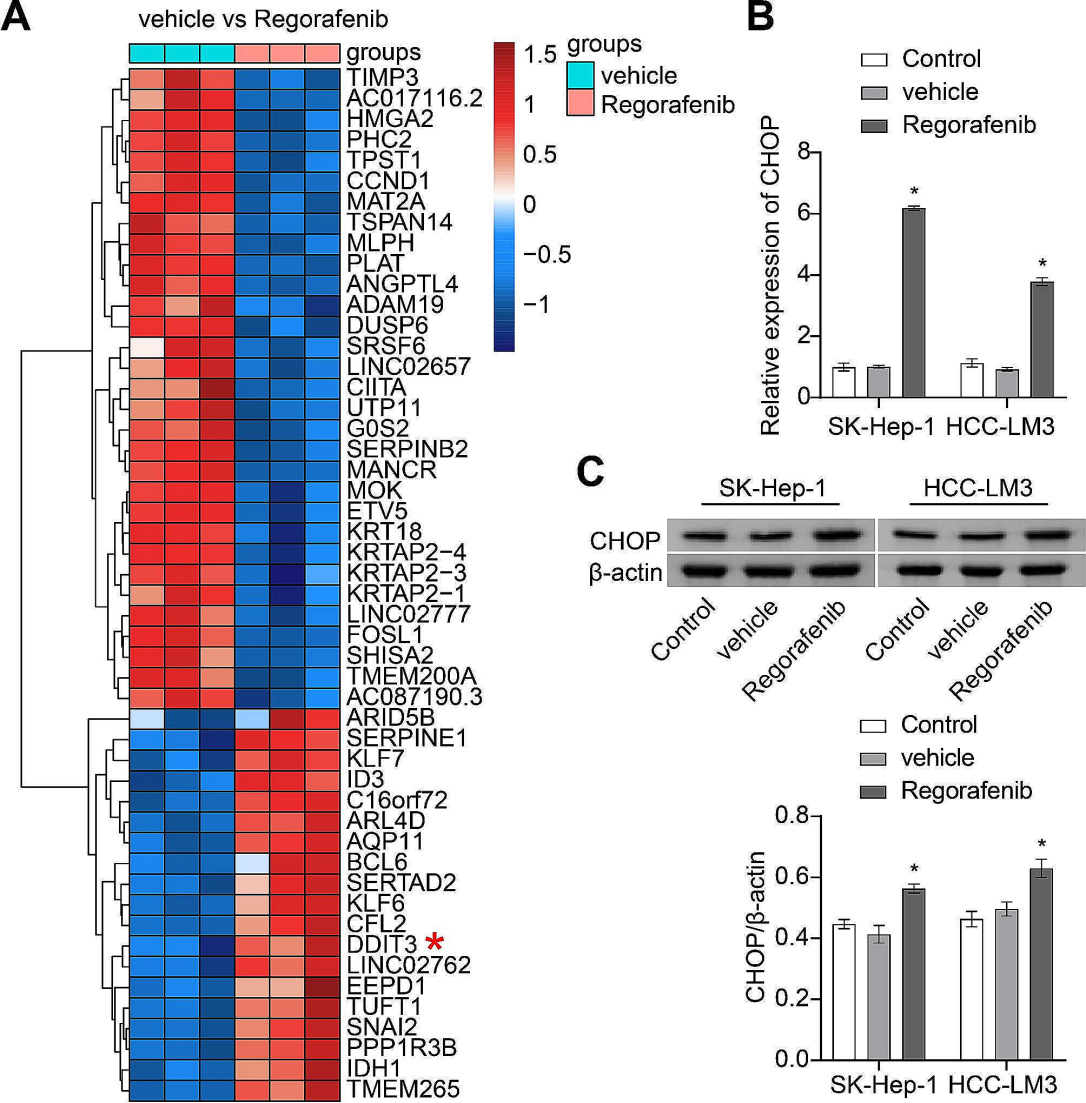
Regorafenib intervention upregulates CHOP mRNA expression in HCC cells. (**A**) Heatmap of differentially expressed mRNAs resulting from Regorafenib intervention. (**B**) Validation of CHOP mRNA expression in SK-Hep-1 and HCC-LM3 cells using RT-qPCR. *P < 0.05 vs. vehicle

Sequencing analysis was conducted to evaluate the mRNA expression profile in SK-Hep-1 cells following intervention with vehicle and Regorafenib, respectively. In comparison to the vehicle group, the results revealed that Regorafenib intervention upregulated the mRNA expression of DDIT3 (CHOP) in SK-Hep-1 cells (Fig. 2A). To validate these results, the expression of CHOP was assessed in SK-Hep-1 and HCC-LM3 cells after intervention with vehicle and Regorafenib, respectively. Consistent with the results in Fig. 2A, Regorafenib intervention upregulated the expression of CHOP in SK-Hep-1 and HCC-LM3 cells (Fig. 2B-C). Additionally, Regorafenib could also influence the other endoplasmic reticulum stress indicators. Following the Regorafenib intervention, endoplasmic reticulum stress indicators HSPA5 and ATF6 also showed an increasing trend (Figure S2). However, the upward trend of HSPA5 and ATF6 was not as significant as the increase observed in CHOP. In conclusion, Regorafenib intervention could influence the expression profile of HCC cells.

### METTL14-mediated m6A methylation suppresses CHOP expression

**Fig. 3:**
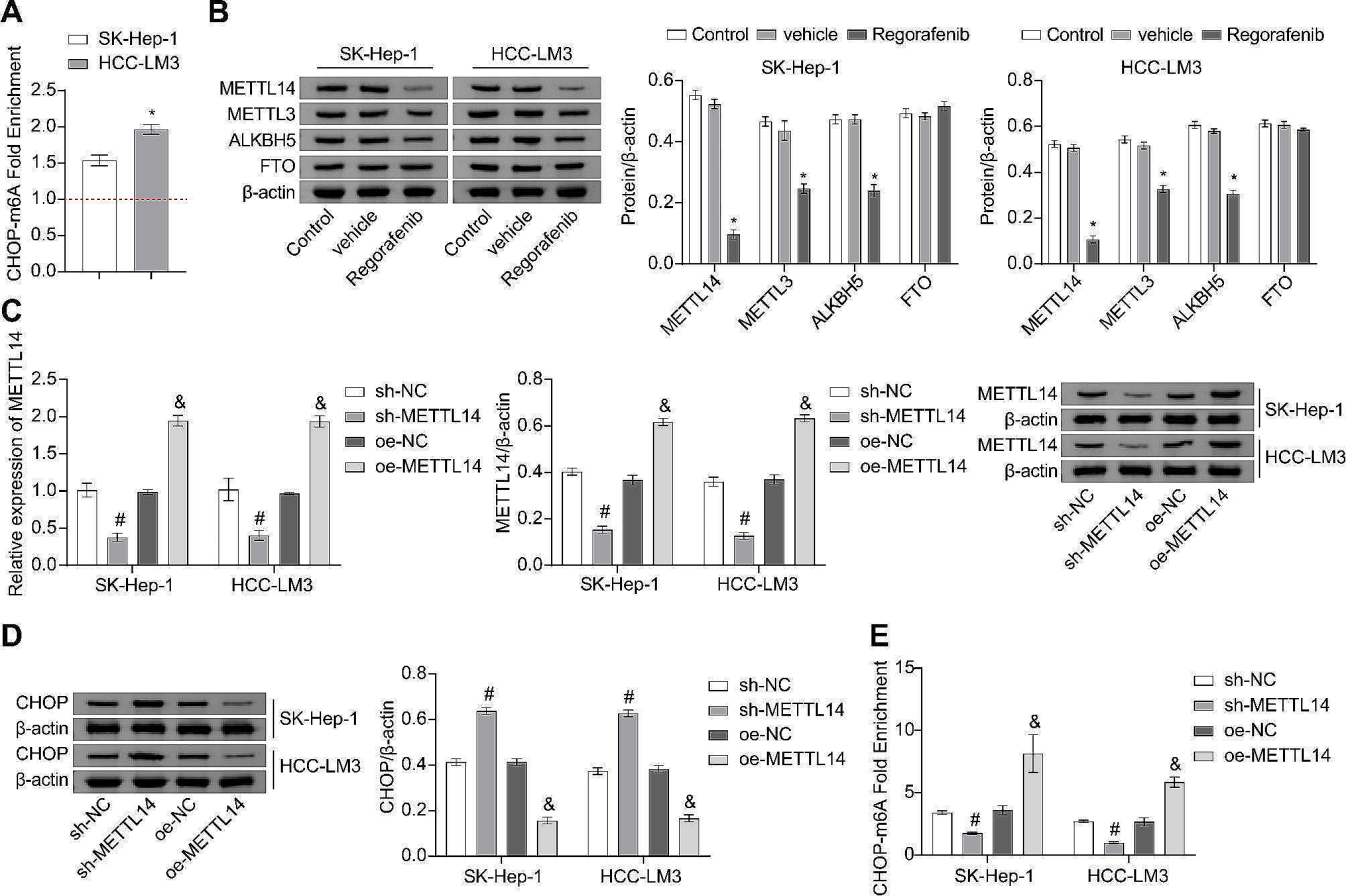
METTL14-mediated m6A methylation suppresses CHOP expression. (**A**) RIP assay for detecting the m6A methylation levels of CHOP. (**B**) WB analysis for the expression of METTL3, METTL14, FTO, and ALKBH5 in SK-Hep-1 and HCC-LM3 cells. (**C-D**) RT-qPCR and WB analysis for the expression of METTL14 and CHOP. (**E**) RIP assay for detecting the m6A levels of CHOP. * *P* < 0.05 vs. vehicle, #*P* < 0.05 vs. sh-NC, &*P* < 0.05 vs. oe-NC

To verify whether CHOP undergoes m6A methylation in HCC cells, RIP was performed on the two cells. Results revealed that the enrichment fold in Fig. 3A is greater than 1, indicating that m6A methylation modification occurred on CHOP mRNA in both SK-Hep1 and HCC-LM3 cells (Fig. 3A). To investigate the protein levels associated with m6A methylation, WB analysis was performed to assess their expression. Following the Regorafenib intervention, the expression of METTL14, METTL3, and ALKBH5 was downregulated in both cells, while the expression of FTO showed no significant change. Among the downregulated proteins, METTL14 exhibited the most significant alteration (Fig. 3B). To further investigate the impact of METTL14-mediated m6A methylation on the expression of CHOP, both cells were subjected to METTL14 knockdown and overexpression treatments. The downregulation of METTL14 expression following sh-METTL14 transfection and the upregulation of METTL14 expression following oe-METTL14 transfection demonstrated the successful and efficient manipulation of METTL14 levels (Fig. 3C). In the subsequent experiments, sh-METTL14 transfection increased CHOP expression and a decrease in CHOP-m6A levels in both SK-Hep-1 and HCC-LM3 cells. Conversely, following oe-METTL14 transfection, a decrease in CHOP expression and an increase in CHOP-m6A levels were observed in both cell lines (Fig. 3D and E). Additionally, the knockdown of METTL3 led to an increase in CHOP expression, which is consistent with the trend observed in Fig. 3D after METTL14 knockdown (Figure S3). These results indicate that the expression of CHOP is regulated by METTL14-mediated m6A methylation.

### Regulation of the cell cycle arrest by Regorafenib through METTL14-mediated modulation of CHOP

**Fig. 4:**
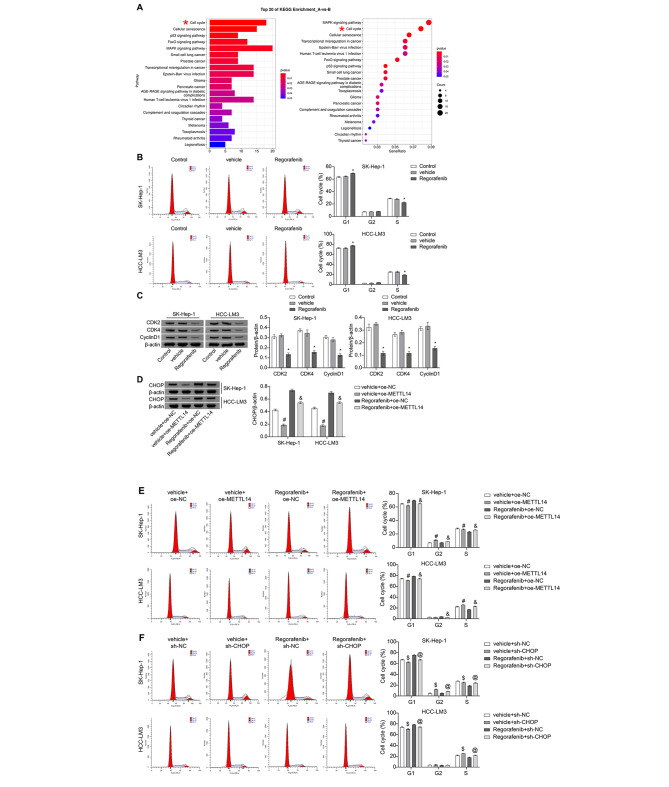
Regulation of the cell cycle arrest by Regorafenib through METTL14-mediated modulation of CHOP. (**A**) KEGG functional enrichment analysis. (**B**) FCM analysis of the cell cycle. (**C**) WB analysis of the expression of CDK2, CDK4, and CyclinD1. (**D**) WB analysis of CHOP expression. (**E-F**) FCM analysis of the cell cycle. * *P* < 0.05 vs. vehicle, # *P* < 0.05 vs. vehicle + oe-NC, & *P* < 0.05 vs. Regorafenib + oe-NC, $ *P* < 0.05 vs. vehicle + sh-NC, @ *P* < 0.05 vs. Regorafenib + sh-NC

Investigating the effect of regorafenib intervention on HCC functionality through bioinformatics analysis, the KEGG pathway analysis revealed that Regorafenib intervention affects cell cycle regulation in HCC cells (Fig. 4A). Following Regorafenib intervention, SK-Hep-1 and HCC-LM3 cells exhibited an elongated G1 phase and a shortened S phase (Fig. 4B). Subsequently, WB analysis of cell cycle-related proteins revealed that the expression of CDK2, CDK4, and Cyclin D1 were all suppressed by Regorafenib intervention in both SK-Hep-1 and HCC-LM3 cells (Fig. 4C). To investigate the specific regulatory mechanism of Regorafenib intervention on the cell cycle, oe-METTL14 transfection was conducted, and was implemented the Regorafenib intervention on the cells. The transfection of oe-METTL14 reduced the promoting impact of Regorafenib intervention on the expression of CHOP in both cell types (Fig. 4D). Furthermore, transfection with oe-METTL14 weakened the cell cycle arrest effect of Regorafenib intervention, characterized by a shortened G1 phase and prolonged S phase (Fig. 4E). In SK-Hep-1 cells, RT-qPCR and WB results indicated that CHOP expression was downregulated following sh-CHOP transfection, while CHOP expression was upregulated following oe-CHOP transfection, demonstrating successful and efficient manipulation of CHOP levels (Figure S1 A-B). Subsequently, sh-CHOP transfection and Regorafenib intervention were performed on both cells. It was observed that transfection with sh-CHOP also attenuated the cell cycle arrest effect of Regorafenib intervention, resulting in a shortened G1 phase and prolonged S phase (Fig. 4F). These results collectively indicated that Regorafenib regulates cell cycle arrest through METTL14-mediated modulation of CHOP.

### The influence of the METTL14/CHOP Axis on the sensitivity of HCC Cells to Regorafenib

**Fig. 5:**
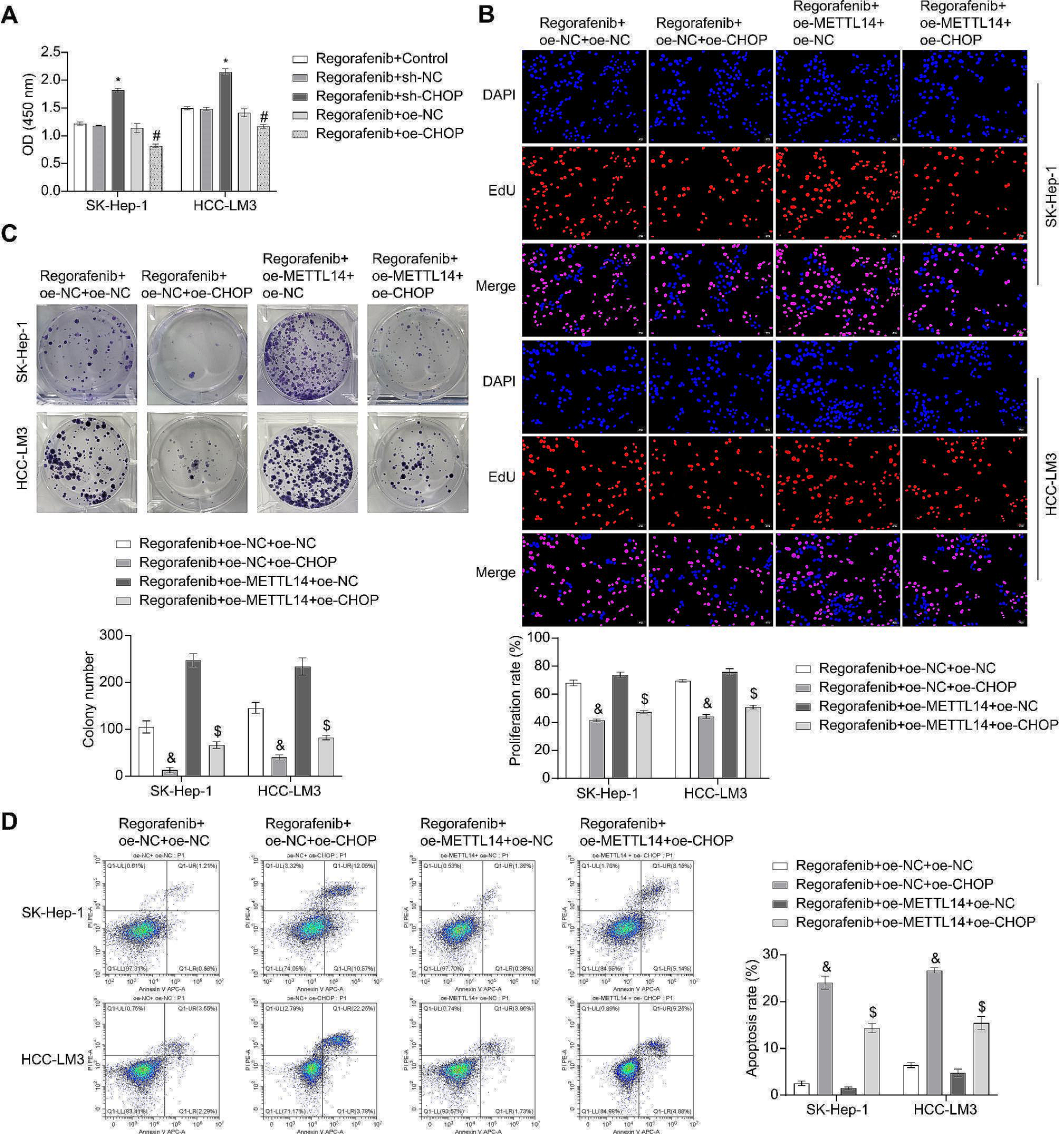
The Influence of the METTL14/CHOP axis on the sensitivity of HCC cells to Regorafenib. (**A**) Assessment of the activity of the cells using the CCK8 assay. (**B**) Evaluation of the proliferation rate of SK-Hep-1 and HCC-LM3 cells using EdU staining. (**C**) Colony formation assay to measure the proliferative activity of SK-Hep-1 and HCC-LM3 cells. (**D**) Detection of the apoptosis rate using FCM. * *P* < 0.05 vs. Regorafenib + sh-NC, # *P* < 0.05 vs. Regorafenib + oe-NC, & *P* < 0.05 vs. Regorafenib + oe-NC + oe-NC, $ *P* < 0.05 vs. Regorafenib + oe-METTL14 + oe-NC

Using CCK8 analysis to assess cell proliferation activity, the results demonstrated that Regorafenib inhibited the viability of SK-Hep-1 and HCC-LM3 cells. However, transfection with sh-CHOP attenuated the inhibitory effect of Regorafenib, while the effect was reversed with oe-CHOP (Fig. 5A). To examine the involvement of the METTL14/CHOP axis in the Regorafenib treatment of HCC, the cells were transfected with oe-METTL14 and oe-CHOP, followed by Regorafenib intervention. The results showed that oe-CHOP transfection enhanced the effect of Regorafenib intervention, leading to decreased proliferative activity, reduced colony formation, and increased apoptosis in both cells. However, oe-METTL14 attenuated the promoting effect of oe-CHOP transfection on Regorafenib intervention, resulting in increased proliferative activity, enhanced colony formation, and decreased apoptosis in both cells (Fig. 5B and D). These findings suggested that the METTL14/CHOP axis could affect the sensitivity of HCC to Regorafenib and subsequently impact the growth of HCC.

### Enhancement of the anticancer effect of Regorafenib by CHOP

**Fig. 6:**
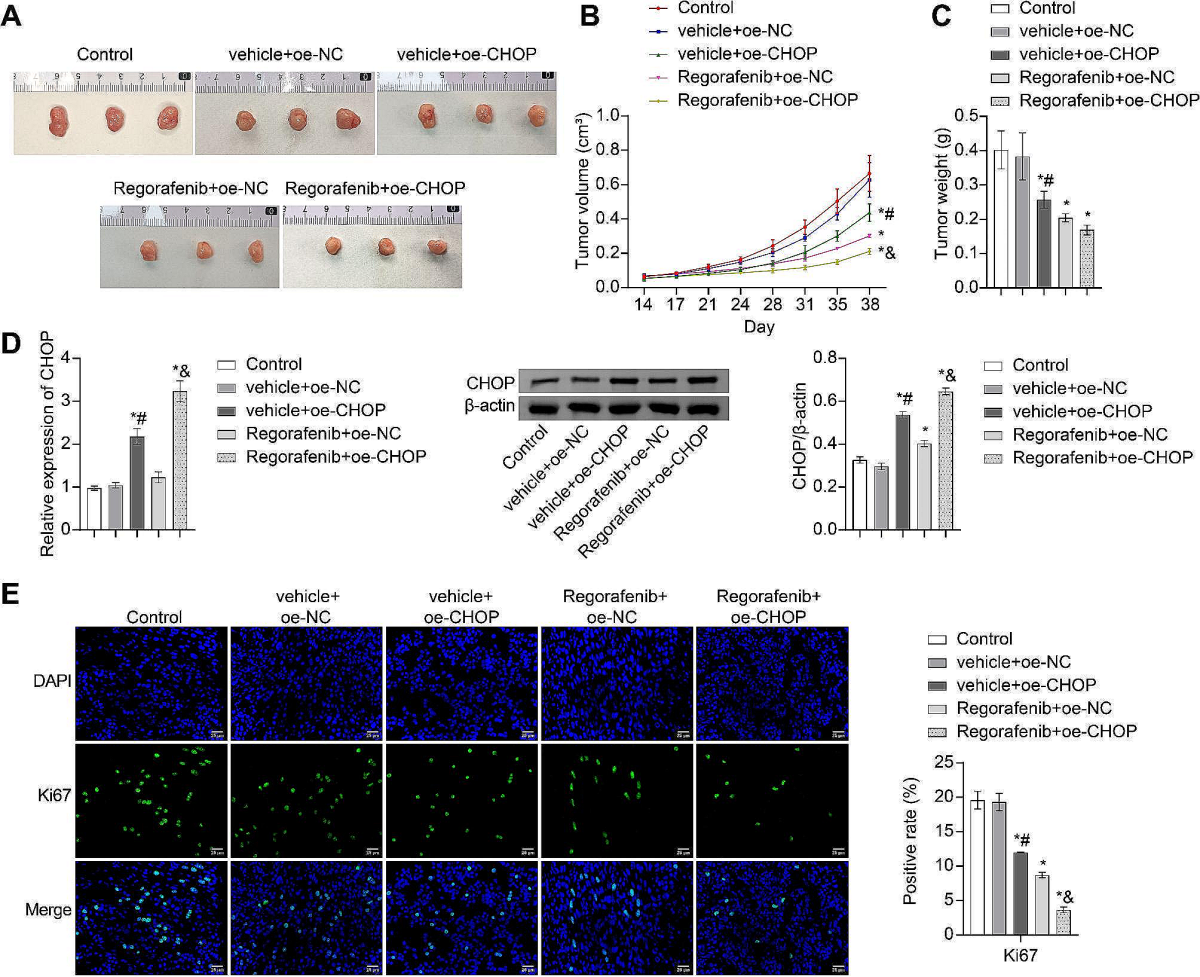
Enhancement of the anticancer effect of Regorafenib by CHOP. (**A**) Tumor images. (**B**) Tumor volume change curve. (**C**) Tumor weight. (**D**) RT-qPCR and WB analysis of CHOP expression in tumors. (**E**) IF staining to detect Ki67 expression in tumors. * *P* < 0.05 vs. Control, # *P* < 0.05 vs. vehicle + oe-NC, & *P* < 0.05 vs. Regorafenib + oe-NC

To validate the in vivo impact of CHOP on the anticancer effect of Regorafenib, a xenograft tumor model was established using HCC-LM3 cells and then transfected with oe-NC or oe-CHOP, followed by intervention with either vehicle or Regorafenib. In comparison to the control group, the Regorafenib + oe-NC group exhibited significant inhibition of cancer growth, with reduced tumor volume and significantly lower tumor mass. Additionally, compared to the Regorafenib + oe-NC group, the Regorafenib + oe-CHOP group showed even lower tumor volume and mass (Fig. 6A and C). Based on our RT-qPCR and WB analysis, it was observed that the expression of CHOP was significantly increased in the tumors of the vehicle + oe-NC group, as compared to the Control group. The expression of CHOP was further increased in the tumors of the Regorafenib + oe-CHOP group in comparison to the Regorafenib + oe-NC group. (Fig. 6D). Additionally, the IF staining results indicated that compared to the Control group, the expression of Ki67 was downregulated in the tumors of the Regorafenib + oe-NC group. Furthermore, compared to the Regorafenib + oe-NC group, the expression of Ki67 was further reduced in the tumors of the Regorafenib + oe-CHOP group (Fig. 6E). These results collectively indicated that CHOP could enhance the anticancer effect of Regorafenib.

## Discussion

In developing countries, such as China, HCC has emerged as the second most prevalent cause of cancer-related deaths among males [30]. Despite progress in the early detection and specialized treatment methods for HCC, patients continue to face challenges such as poor prognosis and high rates of tumor recurrence [31]. It remains a topic of significant interest in clinical research, warranting the exploration of new therapeutic targets. Regorafenib, an orally administered targeted therapy with a broad spectrum of action, has shown efficacy in treating multiple cancer types [32, 33]. While Arai *et al.* have evaluated the safety and effectiveness of Regorafenib, the specific mechanism underlying its action remains unclear [34]. Currently, research on Regorafenib in cancer primarily relies on clinical experiments, with limited studies investigating its specific mechanisms, predominantly in the context of colorectal cancer. We investigated to explore the impact of Regorafenib on HCC and elucidated its underlying mechanism, thereby contributing to the understanding of its therapeutic potential.

Following a similar research approach to Subramonian *et al.*, who investigated the treatment of neuroblastoma with Regorafenib, we conducted screenings using multiple cancer cell lines to identify Regorafenib-sensitive strains and determine the optimal intervention concentration [17]. Four cell lines were selected for screening, revealing that SK-Hep-1 and HCC-LM3 cell lines exhibited higher sensitivity to Regorafenib. Notably, Regorafenib demonstrated a significant inhibitory effect on cancer cell proliferation, echoing the findings reported by Subramanian *et al.* [17].

Subsequently, we investigated the mechanism underlying Regorafenib’s action in HCC. Research findings have shown the involvement of CHOP in the regulation of the hypoxic mechanism in HCC cells [21]. Moreover, we discovered an upregulation of CHOP expression through mRNA sequencing analysis following the administration of Regorafenib. Further, RIP experiments unveiled the m6A methylation modification on CHOP in HCC. m6A methylation serves as a prevalent epigenetic modification, with METTL14 acting as a key writer protein that enhances m6A methylation levels [27, 28]. Notably, in renal cancer, METTL14 suppresses the migration and invasion capabilities of renal cancer cells through m6A modification [35]. Our research uncovered a significant downregulation of METTL14 expression following Regorafenib intervention. Knockdown of METTL14 through shRNA transfection induced a significant reduction in m6A levels of CHOP mRNA, concomitant with an upregulation of CHOP expression levels. According to reports, METTL14 has been shown to promote the decay of CHOP mRNA through m6A methylation modification [22]. These findings suggested that Regorafenib can promote the expression of CHOP by weakening the m6A methylation mediated by METTL14. Wei *et al.* found that METTL14-mediated m6 modification inhibits the expression of CHOP, and our results are consistent with theirs [22].

Regorafenib intervention has been shown to induce apoptosis and cell cycle arrest in cancer cells [17]. Moreover, our study elucidated that Regorafenib intervention exerted notable effects on cell cycle progression in the cells (SK-Hep-1 and HCC-LM3), leading to a prominent extension of the G1 phase alongside a concurrent reduction in the S phase. Importantly, transfection with sh-CHOP prominently mitigated the effects of Regorafenib intervention. Our results indicated that Regorafenib exerts effects on cell cycle arrest in cancer cells through the regulation of CHOP via the METTL14-m6A pathway. Consistent with the findings reported by Subramonian *et al.*, Regorafenib treatment was observed to significantly lengthen the G1 phase, while transfection of CHOP plasmids induced G1/S arrest in proliferating NIH3T3 cells [17, 36]. Our findings corroborated these studies’ conclusions. In our subsequent experiments, we observed that transfection with oe-CHOP significantly increased sensitivity to regorafenib, leading to a decrease in cell proliferation activity, reduced clone formation, and an increase in apoptosis in the cells. On the other hand, oe-METTL14 weakened the enhancing effect of oe-CHOP transfection on regorafenib intervention. Our findings provided compelling evidence that the METTL14/CHOP axis significantly influences the sensitivity of HCC cells to Regorafenib. In vivo, our findings showed that the enhancement of CHOP significantly potentiated the anticancer effects of Regorafenib, resulting in a notable reduction in tumor volume and mass in mice, which is consistent with our in vitro cell experiment results. In contrast to Fang study, which did not provide a clear explanation for how ATF4 and ATF3 regulate CHOP to induce apoptosis in human lens epithelial cells, our experiments successfully elucidated the mechanism through which Regorafenib regulates the expression of CHOP [37]. We elucidated the mechanism of Regorafenib in regulating CHOP through our experiments and demonstrated that the METTL14/CHOP axis also contributes to the effects of Regorafenib.

However, this study presents several limitations that should be acknowledged. Specifically, our research was limited to animal experiments and did not include validation of the effectiveness in clinical settings.

## Conclusions

Our research findings provide evidence supporting the effectiveness of Regorafenib against both HCC cell lines and xenograft tumors, highlighting the potential of Regorafenib as a promising therapeutic intervention for HCC. Regorafenib could regulate the m6A expression of CHOP through METTL14 to impact cell cycle arrest in HCC cells. In addition, the METTL14/CHOP axis could also influence the sensitivity of Regorafenib. In conclusion, our research results confirm the involvement of the METTL14-m6A mechanism in the regulation of CHOP-mediated cell cycle arrest in hepatocellular carcinoma. This mechanism influences the sensitivity of Regorafenib and impedes the growth of HCC. Our research provides insights into the therapeutic implications of HCC and the issue of drug resistance to Regorafenib.

### Electronic supplementary material

Below is the link to the electronic supplementary material.

Supplementary Material 1

## Data Availability

The data used in this study are included in the paper. Raw data can be obtained from the corresponding author upon reasonable request. The original mRNA sequencing data has been uploaded to the NCBI database, which can be obtained at the link: https://www.ncbi.nlm.nih.gov/sra/PRJNA1056523.
