# Supplementary material for: CHOP regulated by METTL14-m6A affects cell cycle arrest and regorafenib sensitivity in HCC cells
Source: BMC Cancer. 2024 Apr 25;24:525. doi: 10.1186/s12885-024-12275-w (PMC11046807; doi:10.1186/s12885-024-12275-w)
Supplement: Supplementary file 1 — Supplementary Material 1 [file 12885_2024_12275_MOESM1_ESM.docx]

**Supplementary materials**

**
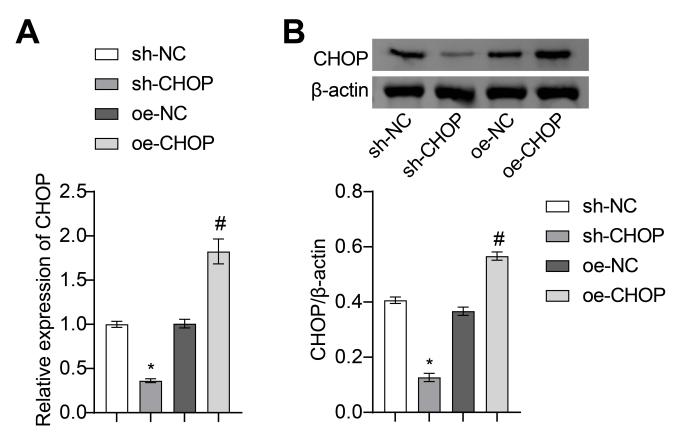
**

**Figure S1. Detection of CHOP Expression in SK-Hep-1 Cells Using RT-qPCR and WB.** (A) RT-qPCR analysis for the expression of CHOP in SK-Hep-1 Cells. (B) WB analysis for the expression of CHOP in SK-Hep-1 Cells. * P<0.05 vs. sh-NC, # P<0.05 vs. oe-NC.


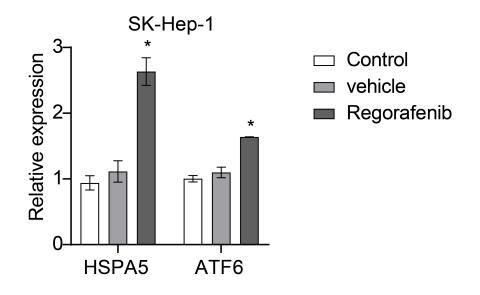


**Figure S2. Regorafenib promotes the expression of endoplasmic reticulum stress markers HSPA5 and ATF6.** RT-qPCR analysis for the expression of HSPA5 and ATF6 in SK-Hep-1 Cells.


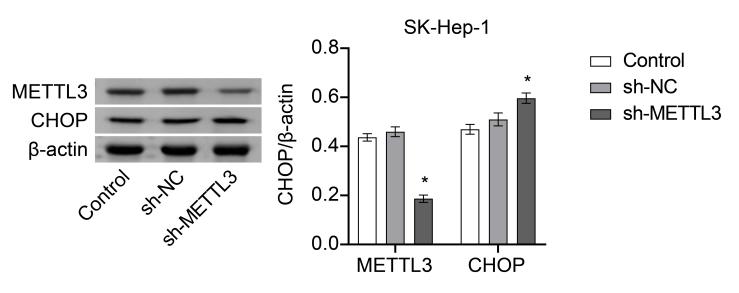


**Figure S3.** **Knockdown of** **METTL3 promotes the expression of CHOP.** WB analysis for the expression of METTL3 and CHOP in SK-Hep-1 Cells.


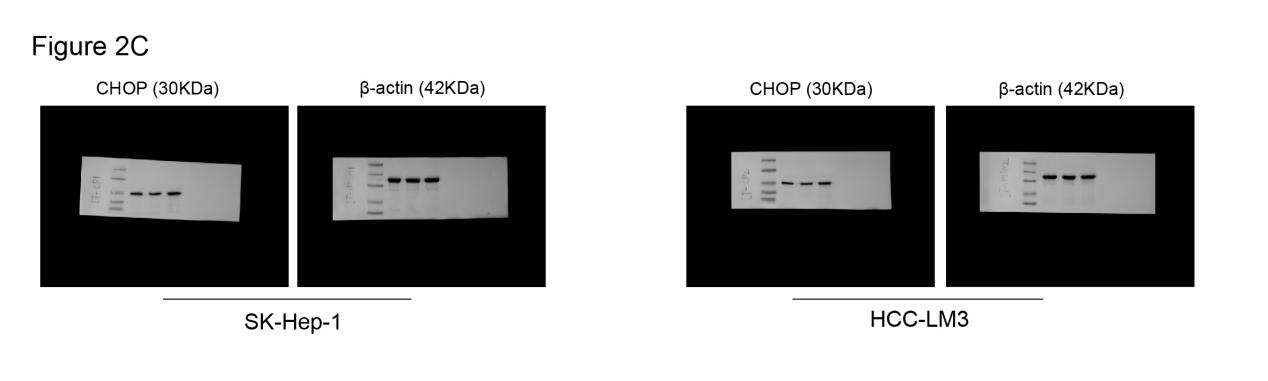


**Figure S4. Full uncropped Blots images of Figure 2**

**
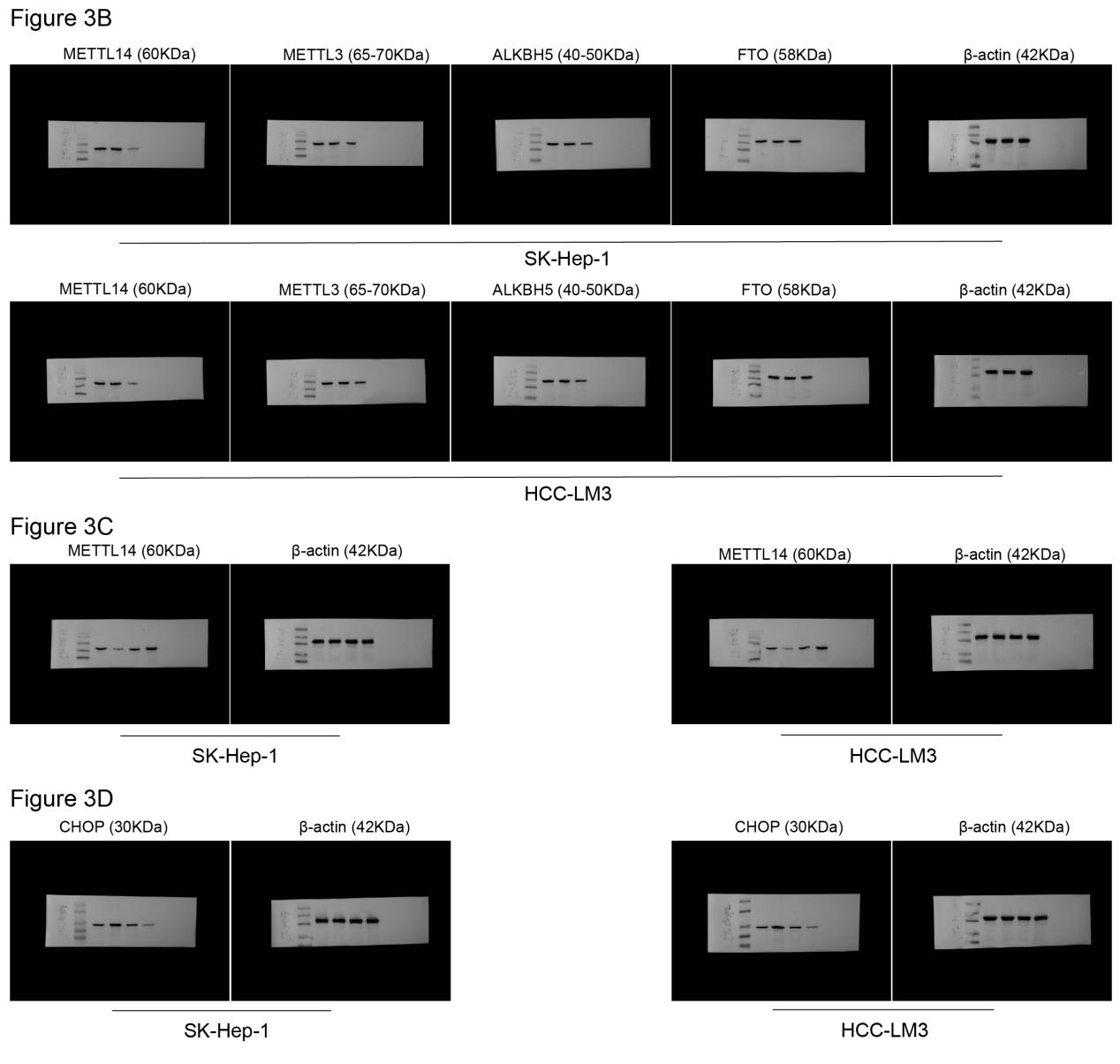
**

**Figure S5. Full uncropped Blots images of Figure 3**

**
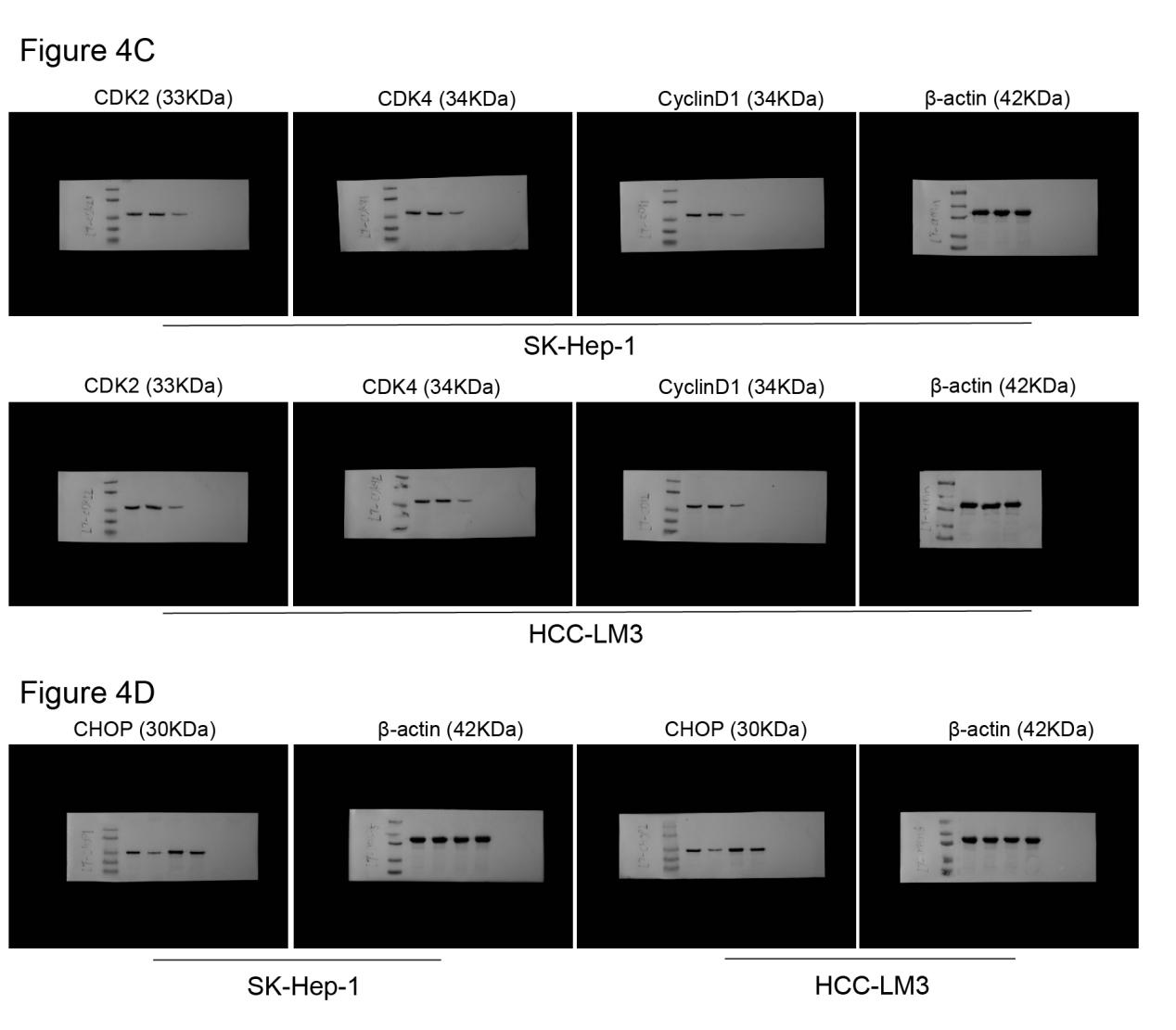
**

**Figure S6. Full uncropped Blots images of Figure 4**

**
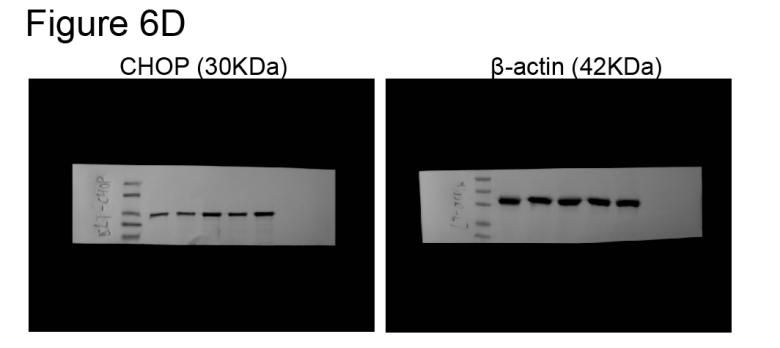
**

**Figure S7. Full uncropped Blots images of Figure 6**

**
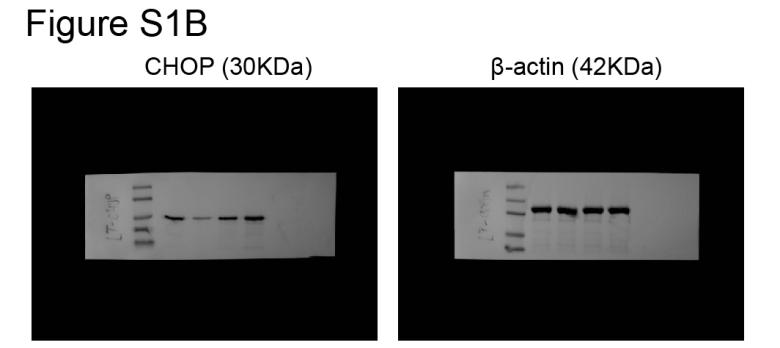
**

**Figure S8. Full uncropped Blots images of Figure S1**

**
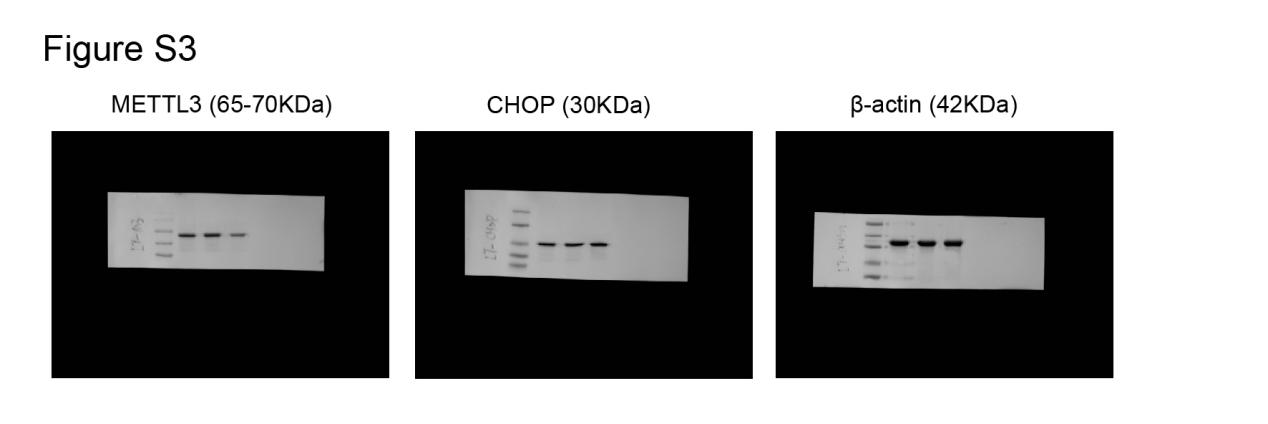
**

**Figure S9. Full uncropped Blots images of Figure S3**
